# Idiopathic Retroperitoneal Fibrosis Mimicking Malignancy and Presenting With Obstructive Uropathy and Acute Kidney Injury: A Case Report

**DOI:** 10.7759/cureus.102534

**Published:** 2026-01-29

**Authors:** André Calheiros, Patrícia Araújo, Carlos Gonçalves, Nuno Pardal, Nereida Monteiro

**Affiliations:** 1 Internal Medicine, Unidade Local de Saúde do Alto Minho, Hospital Conde de Bertiandos, Ponte de Lima, PRT; 2 Department of Internal Medicine, Unidade Local de Saúde do Alto Minho, Hospital de Santa Luzia, Viana do Castelo, PRT; 3 Medicine, Unidade Local de Saúde do Alto Minho, Hospital Conde de Bertiandos, Ponte de Lima, PRT; 4 Internal Medicine, Unidade Local de Saúde do Alto Minho, Hospital de Santa Luzia, Viana do Castelo, PRT

**Keywords:** idiopathic retroperitoneal fibrosis, obstructive uropathy, retroperitoneal fibrosis, retroperitoneal mass, ureteral obstruction

## Abstract

Retroperitoneal fibrosis is a rare fibroinflammatory disorder that can lead to progressive encasement of retroperitoneal structures, including the aorta, inferior vena cava, ureters, and iliac vessels, most commonly resulting in ureteral obstruction.

It may be idiopathic or secondary to medications, malignancy, radiotherapy, or other causes. Its radiological appearance often mimics malignant disease, which may complicate the diagnostic approach, particularly in patients with a previous oncologic history.

We report the case of a 70-year-old man with a past medical history significant for chronic lymphocytic leukemia under surveillance and intermediate-risk prostate adenocarcinoma previously treated with hormone therapy and currently without evidence of active disease, who presented to the emergency department with persistent left-sided flank pain.

Contrast-enhanced abdominal computed tomography revealed an irregular left retroperitoneal soft-tissue density, measuring approximately 8 cm in longitudinal extent, located anteroinferior to the aortic bifurcation, encasing the left ureter and causing mild to moderate ipsilateral hydronephrosis.

Given concern for malignancy, a biopsy of the retroperitoneal lesion and an adjacent lymph node was performed, demonstrating fibro-adipose tissue with a mononuclear inflammatory infiltrate and no evidence of neoplasia. Serum immunoglobulin G4 levels were within normal limits.

After exclusion of alternative diagnoses, corticosteroid therapy was initiated, resulting in rapid clinical improvement and complete radiological resolution on follow-up imaging.

Retroperitoneal fibrosis should be considered in patients presenting with unexplained obstructive uropathy, and an etiological workup is essential to exclude secondary causes and guide appropriate management.

## Introduction

Retroperitoneal fibrosis is a rare fibroinflammatory disorder characterized by the development of dense fibrotic tissue in the retroperitoneum, leading to progressive encasement of adjacent structures, most commonly the ureters and great vessels. This process frequently results in obstructive uropathy and presents with nonspecific symptoms, often delaying diagnosis [1]. It is an uncommon condition, with an estimated incidence ranging from approximately 0.1 to 1.3 cases per 100,000 persons per year [2]. Current evidence suggests that idiopathic retroperitoneal fibrosis is increasingly recognized as an immune-mediated fibroinflammatory process, characterized by chronic inflammation and progressive fibrosis of retroperitoneal tissues [3].

The condition may be idiopathic or secondary to malignancy, radiotherapy, medications, infections, or systemic inflammatory and autoimmune diseases. A subset of cases is associated with immunoglobulin G4-related disease, highlighting the importance of a structured etiological evaluation [4].

On imaging, retroperitoneal fibrosis typically appears as a soft-tissue mass surrounding the great vessels and ureters, an appearance that may closely mimic malignant retroperitoneal disease. This overlap poses a significant diagnostic challenge, particularly in patients with a prior oncologic history, and often necessitates histopathological confirmation [1,5].

We report a case of idiopathic retroperitoneal fibrosis presenting with obstructive uropathy in a patient with previous malignancy, emphasizing the diagnostic approach, the role of biopsy in excluding neoplasia, and the favorable response to corticosteroid therapy.

## Case presentation

A 70-year-old man presented to the emergency department with a three-week history of progressive left-sided flank pain, associated with nausea but no vomiting. He denied fever, urinary symptoms, weight loss, or other constitutional complaints. His past medical history was significant for chronic lymphocytic leukemia (Rai stage I), diagnosed in 2019 and managed with active surveillance, and intermediate-risk prostate adenocarcinoma treated with androgen deprivation therapy approximately two years prior to presentation, currently under surveillance with no evidence of active disease at the time of presentation.

On physical examination, the patient was hemodynamically stable and afebrile. Abdominal examination revealed no palpable masses and no costovertebral angle tenderness. The remainder of the physical examination was unremarkable.

Initial laboratory evaluation revealed acute kidney injury, with a serum creatinine level of 1.82 mg/dL compared to a known baseline value of 0.89 mg/dL, along with elevated inflammatory markers, including C-reactive protein (7 mg/dL) and erythrocyte sedimentation rate (68 mm/h). Complete blood count showed no significant abnormalities. Urinalysis was unremarkable, with no hematuria, proteinuria, leukocyturia, or evidence of urinary tract infection.

**Figure 1:**
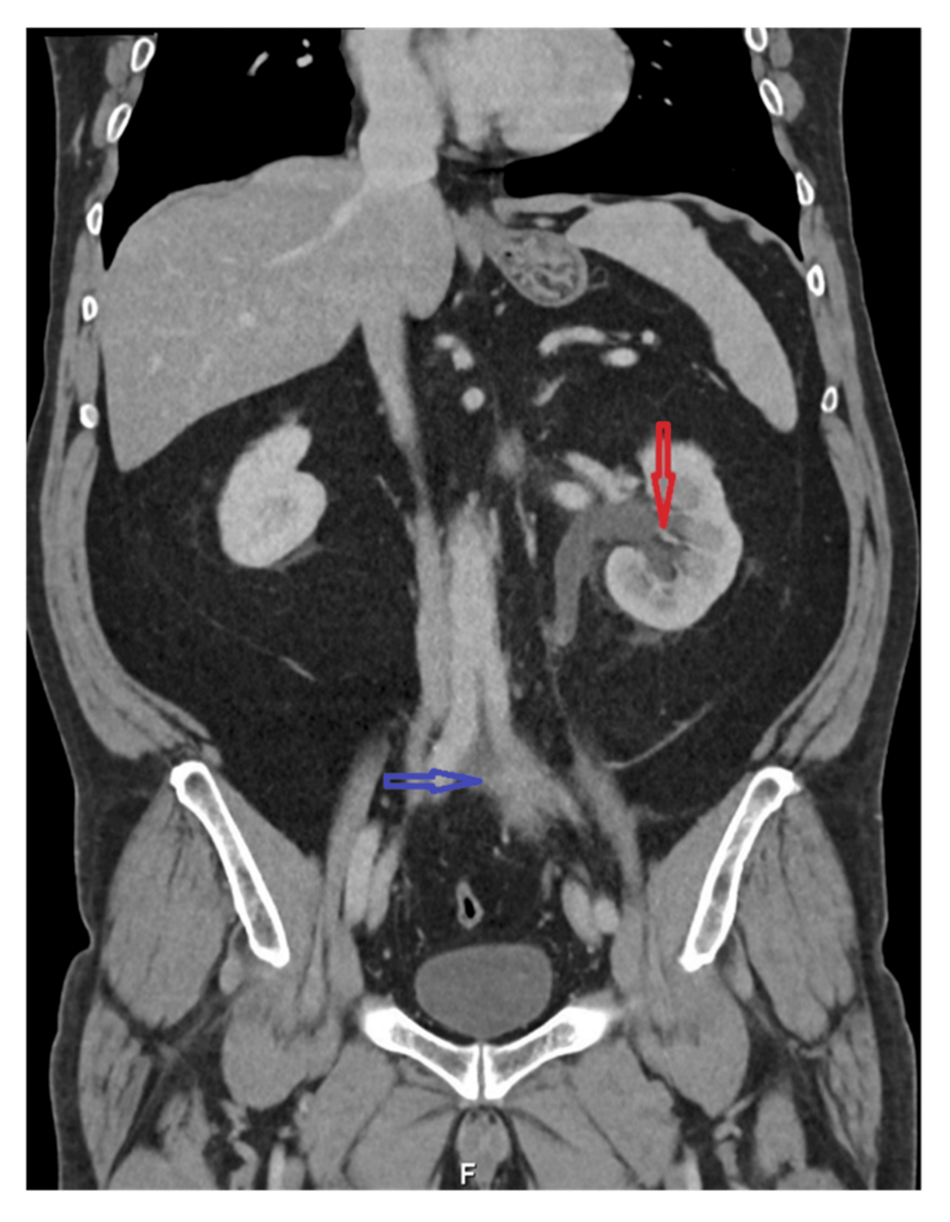
Abdominal and pelvic contrast-enhanced CT Contrast-enhanced abdominal computed tomography (coronal view) showing irregular left-sided retroperitoneal soft-tissue thickening anterior to the left common iliac vessels (blue arrow), associated with mild to moderate ipsilateral hydronephrosis (red arrow).

Contrast-enhanced abdominal computed tomography demonstrated an irregular left-sided retroperitoneal soft-tissue mass, measuring approximately 8 cm in longitudinal extent, located anteroinferior to the aortic bifurcation. The lesion encased the left ureter, resulting in mild to moderate ipsilateral hydronephrosis, without evidence of distant lymphadenopathy or metastatic disease (Figure 1).

On the day of admission, given the presence of obstructive uropathy and acute kidney injury, a left percutaneous nephrostomy was performed, leading to prompt improvement in renal function. Considering the patient’s prior oncologic history and the radiological appearance of the lesion, malignancy remained a concern. A CT-guided biopsy of the retroperitoneal mass and an adjacent lymph node was therefore undertaken. Histopathological examination revealed fibro-adipose tissue with a mononuclear inflammatory infiltrate and no evidence of malignant cells.

A targeted etiological workup was performed. There was no clinical or laboratory evidence of active malignancy, infection, or systemic autoimmune disease. Serum immunoglobulin G4 levels were within normal limits, and no medications previously associated with secondary retroperitoneal fibrosis were identified. As part of the etiological and staging evaluation, fluorodeoxyglucose positron emission tomography demonstrated mild glycolytic uptake in the soft-tissue thickening along the course of the left ureter, most evident at the L5 level, interpreted as likely inflammatory in nature, with no other abnormal hypermetabolic findings.

After exclusion of secondary causes, a diagnosis of idiopathic retroperitoneal fibrosis was established. Corticosteroid therapy was initiated with oral prednisone at a dose of 60 mg/day (approximately 0.75 mg/kg/day). The initial full dose was maintained for four weeks, followed by a gradual tapering regimen over the subsequent months, for a total treatment duration of approximately six months. The patient experienced rapid clinical improvement, with resolution of flank pain and normalization of renal function.

**Figure 2:**
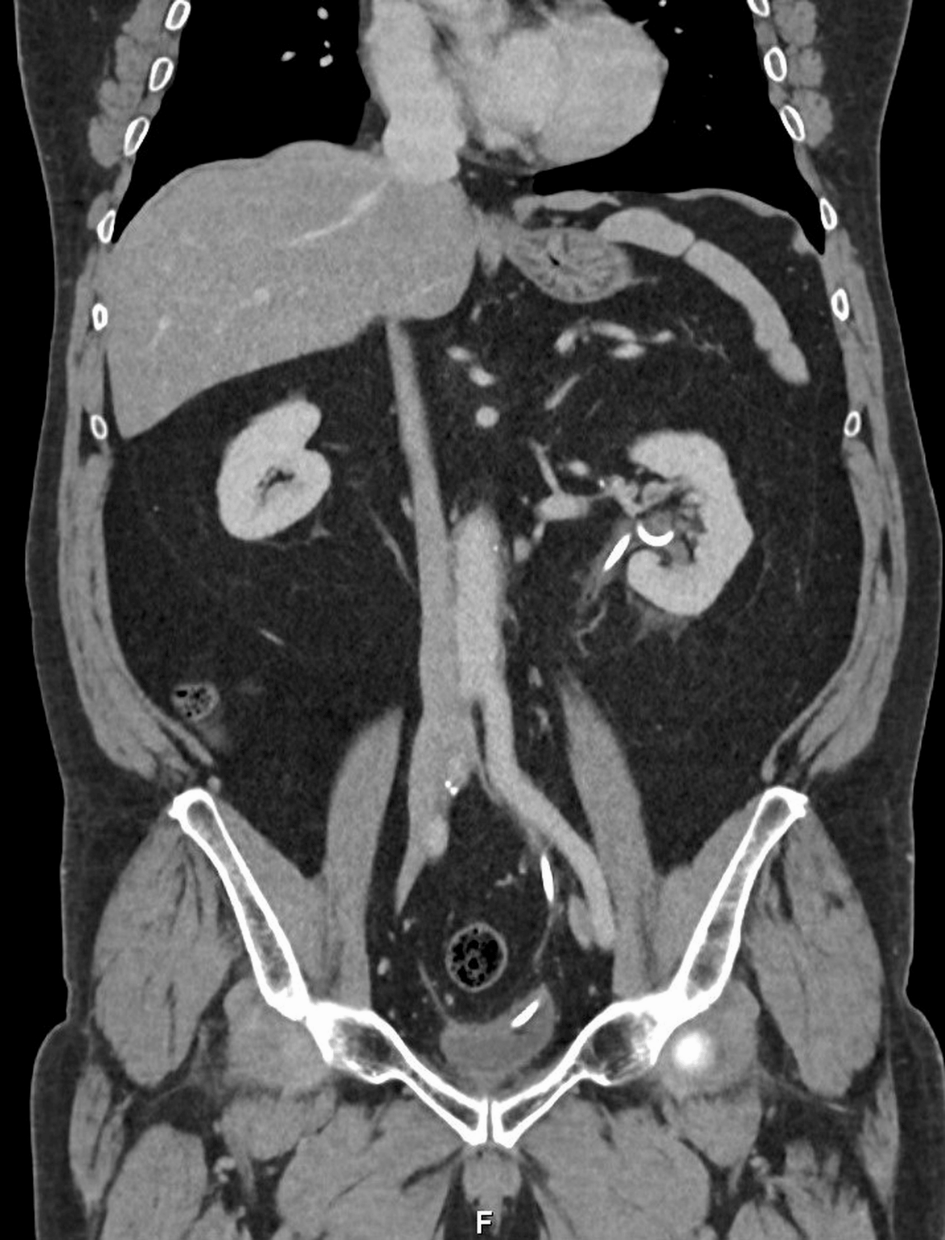
Follow-up abdominal and pelvic contrast-enhanced CT Follow-up contrast-enhanced abdominal and pelvic computed tomography (coronal view) demonstrating complete resolution of the previously described retroperitoneal soft-tissue thickening, with no residual ureteral obstruction.

During follow-up, the percutaneous nephrostomy was successfully removed and replaced with a left double-J ureteral stent, without complications. Follow-up contrast-enhanced abdominal computed tomography demonstrated complete resolution of the retroperitoneal fibrotic mass, with no residual ureteral obstruction (Figure 2). At the six-month follow-up, the patient had completed corticosteroid tapering one week prior, remained asymptomatic, and had stable renal function with serum creatinine within the normal range, along with normalization of inflammatory markers (C-reactive protein: 0.8 mg/dL; erythrocyte sedimentation rate: 12 mm/h).

## Discussion

Idiopathic retroperitoneal fibrosis is an uncommon fibroinflammatory condition that often presents with nonspecific symptoms and may lead to delayed diagnosis. Obstructive uropathy is the most frequent clinical manifestation and can result in acute kidney injury, as observed in this case. Because radiological findings frequently overlap with malignant retroperitoneal disease, particularly in patients with a prior oncologic history, establishing the correct diagnosis can be challenging. In this case, the presence of elevated inflammatory markers at presentation further supported an active fibroinflammatory process [1]. Recent reviews have emphasized the evolving understanding of retroperitoneal fibrosis as an immune-mediated fibroinflammatory disease, with important implications for diagnosis and management [3].

Malignancy represents a key differential diagnosis, especially in patients with known or previous cancer, as retroperitoneal neoplasms and lymphoproliferative disorders may present with similar imaging features. In such cases, histopathological confirmation is often required to exclude neoplasia and avoid inappropriate oncologic treatment [1,5]. Several reports have described idiopathic retroperitoneal fibrosis presenting in patients with a prior history of malignancy, highlighting the diagnostic dilemma and reinforcing the importance of tissue confirmation in this context [1,5].

Another important consideration is immunoglobulin G4-related disease, which accounts for a subset of cases previously classified as idiopathic retroperitoneal fibrosis. Assessment of serum immunoglobulin G4 levels and histopathological features is therefore recommended as part of the etiological evaluation [4]. In the present case, histopathological examination demonstrated fibro-adipose tissue with a prominent mononuclear inflammatory infiltrate and no features typically associated with IgG4-related disease. Immunohistochemical analysis revealed a reactive mixed T- and B-cell population (CD3/CD20), without evidence of malignancy. Given the absence of histopathological features suggestive of IgG4-related disease, immunohistochemical staining specifically for IgG4 was not performed, and serum IgG4 levels were within normal limits. Although normal serum IgG4 levels do not exclude IgG4-related disease, the absence of suggestive histopathological features made this diagnosis unlikely in the present clinical context.

Management of retroperitoneal fibrosis depends on the severity of organ involvement and the presence of complications. In patients presenting with obstructive uropathy and renal impairment, prompt urinary decompression is required to preserve renal function. Corticosteroids remain the first-line treatment for idiopathic retroperitoneal fibrosis and are associated with high rates of clinical and radiological response [6]. While urinary drainage procedures are essential for relieving obstruction and preserving renal function, they do not lead to regression of the fibroinflammatory mass. The complete radiological resolution observed in this case is therefore attributable to corticosteroid therapy. In addition to clinical and radiological improvement, normalization of inflammatory markers provided objective evidence of treatment response.

This case highlights the importance of considering idiopathic retroperitoneal fibrosis in patients presenting with unexplained obstructive uropathy, even in the context of prior malignancy. Rather than representing an exceptionally rare presentation, this case illustrates a diagnostic pitfall in which a benign fibroinflammatory condition may closely mimic malignant disease, particularly in patients with a prior oncologic history. A structured diagnostic approach, including histopathological confirmation when malignancy cannot be excluded, is crucial for guiding appropriate management and achieving favorable outcomes.

## Conclusions

This case describes retroperitoneal fibrosis presenting with obstructive uropathy and acute kidney injury in a patient with a prior oncologic history, initially raising concern for malignant disease. Comprehensive imaging assessment and histopathological confirmation were essential to exclude neoplasia and establish the diagnosis. Following urinary decompression, corticosteroid therapy resulted in rapid clinical improvement, normalization of renal function, inflammatory markers, and radiological resolution. This case illustrates the diagnostic difficulty encountered in a patient with a prior oncologic history and highlights the importance of etiological investigation, given its direct implications for therapeutic decision-making.

As this report describes a single patient, broader conclusions should be interpreted with caution, but the case illustrates the diagnostic challenges associated with retroperitoneal fibrosis in patients with prior malignancy.
